# Were the unfinished nursing care occurrence, reasons, and consequences different between COVID-19 and non-COVID-19 patients? A systematic review

**DOI:** 10.1186/s12912-023-01513-4

**Published:** 2023-09-27

**Authors:** Stefania Chiappinotto, Aysun Bayram, Luca Grassetti, Alessandro Galazzi, Alvisa Palese

**Affiliations:** 1 University of Udine, Udine, Italy; 2 Karadeniz Technical University, Trabzon, Turkey

**Keywords:** Coronavirus-19 pandemic, Systematic review, Unfinished nursing care

## Abstract

**Background:**

Unfinished Nursing Care (UNC) has been documented also during the Coronavirus (COVID-19) pandemic; however, while several secondary studies were conducted before this period to summarise occurrences, reasons, and consequences of UNC and provide a global picture of the phenomenon, no synthesis of the evidence produced during the pandemic has been documented to date. Therefore, the aim of this review is to identify differences, if any, in the UNC occurrence, reasons, and consequences perceived by nurses caring for COVID-19 and non-COVID-19 patients.

**Methods:**

This study is a systematic review (PROSPERO CRD42023410602). According to the Population, Exposure, Comparator, and Outcomes framework, primary comparative cross-sectional, longitudinal, and cohort studies, randomised/non-randomised controlled trials were included from Medline, CINAHL, and Scopus, collecting perceptions of nurses with tools measuring UNC between COVID-19 and non-COVID-19 patients and published in English, Italian, or Turkish. Preferred Reporting Items for Systematic Reviews and Meta-Analyses guideline and Johanna Briggs Quality Appraisal Tool were used, and findings were summarised narratively.

**Results:**

Five hospital-based cross-sectional studies using the self-administered MISSCARE and UNC Survey comparing data collected (a) before the pandemic vs. in the first wave; (b) before, in the second and in the third wave; and (c) simultaneously among COVID-19 and non-COVID-19 patients in the second wave. Three main patterns emerged suggesting a higher UNC occurrence among COVID-19 patients in the first wave, less occurrence among them compared to non-COVID-19 patients in the second wave, and contrasting findings with some in favour and others in contrast to COVID-19 patients. Similar patterns emerged regarding UNC reasons while no studies investigated the UNC consequences.

**Conclusions:**

In the first wave, COVID-19 patients were likely to be at increased risk of UNC, while in later waves non-COVID-19 patients were at increased risk of UNC. Reasons also were different across waves. Findings documented during the COVID-19 pandemic may help to prevent UNC in future disasters.

**Supplementary Information:**

The online version contains supplementary material available at 10.1186/s12912-023-01513-4.

## Background

The quality of nursing care during the coronavirus-19 (COVID-19) pandemic has been reported as critical due to several factors. The huge number of patients requiring care simultaneously [1], the limited competencies of nurses deployed from one unit to another in order to enhance the health service capacity in some units [2], as well as the vulnerability of the same staff reporting the increased number of quarantined nurses [3], have been reported as preventing the delivery of the care required. On the other hand, the limited availability of material resources, mainly in the first wave [4], has been underlined as affecting the quality of care, suggesting an increased occurrence of Unfinished Nursing Care (UNC) [2]. UNC, also known as Task Left Undone [5], Missed Nursing Care [6], or Implicit Rationing of Nursing Care [7], has been documented as the phenomenon in which nurses are not able to ensure the care required by patients by omitting interventions or delaying affecting mainly the fundamental needs of patients (e.g., helping patients to ambulate) (e.g., [8]). Among the reasons, many individual and institutional factors, such as the lack of time or staff and extra working hours, have been identified [9]. As consequences of UNC, negative outcomes for the patient (e.g., pressure sores), nurses (e.g., increased moral distress) (e.g., [10, 11]), and organisational levels (e.g., increased costs due to the increased length of stay) (e.g., [12]) have been reported.

In order to document the occurrence of UNC, quantitative studies have been conducted during the pandemic, reporting higher prevalence among COVID-19 patients [13–15]. Reasons were also documented in some studies as unfavourable environments, overtime work [16], and issues in maintaining adequate staffing levels [14]. In other studies, the additional time required by wearing personal protective equipment (PPE) (e.g., donning and doffing), the severity of patients’ conditions, and the lack of time to spend with patients due to restrictions imposed [15] affecting the communication [17] were recognised as additional factors triggering UNC. Above all, work environment issues [18] characterised by chaos, routine disruption, continuous changes, were underlined in their role triggering UNC.

However, while several secondary studies (e.g., [11, 19]) were conducted before the COVID-19 pandemic to summarise occurrences, reasons, and consequences of UNC and provide a global picture of the phenomenon, no synthesis of the evidence produced during the pandemic has been documented to date. The additional factors documented [15] may have influenced the occurrence of the phenomenon among non-COVID-19 and COVID-19 patients, generating different consequences. Revealing the differences, if any, may increase our understanding of what happened during the pandemic not only among patients affected by COVID-19 but also among those who were at need of care for other health issues. In addition, it may contribute to identify the role of patient profile as a risk factor triggering UNC. Moreover, summarising the differences, if any, in the UNC phenomenon between the two patient groups may also increase our understanding of how to deal with future pandemics by implementing strategies capable of preventing/avoiding issues among both exposed and non-exposed patients. Therefore, to summarise the knowledge developed in this context was the main intent of this study.

## Methods

### Aim

The aim of this study is to identify differences, if any, in the UNC occurrence, reasons, and consequences between COVID-19 and non-COVID-19 patients during the pandemic.

### Design

In a preliminary phase, two researchers (AB, SC) performed a rapid literature search to check the studies, if any, published on UNC during the pandemic period, which started on 11 March 2020 [20]. In line with the retrieved literature, a systematic review research protocol was designed following the Preferred Reporting Items for Systematic Reviews and Meta-Analyses (PRISMA) guideline [21] and then registered in PROSPERO (CRD42023410602). As a result, the following research question was identified: “Did UNC occurrence, reasons, and consequences (hereinafter UNC data) change during the COVID-19 pandemic between COVID-19 and non-COVID-19 patients?”

According to the Population, Exposure, Comparator, and Outcomes (PECO) framework [22], the main criteria of the systematic review were established:

- P, as patients cared for in any setting;
- E, as the exposure, was considered data collected among nurses caring for COVID-19 patients;
- C, as comparisons, was considered UNC data collected among nurses caring for non-COVID-19 patients (a) before the pandemic, in the same context/setting and analysed in the study, and (b) during the pandemic, in different waves;
- O, as the outcome, differences, if any, in the UNC data (occurrence, causes, and consequences) as perceived by nursing staff were considered.

Studies were included that (a) concerned the UNC occurrence, reasons, or consequences in all settings (e.g., hospital, community); (b) were published in English, Italian, or Turkish; (c) collected data from January 1^st^, 2023; (d) involved registered nurses and nursing aides as participants; (e) involved comparative cross-sectional, longitudinal, or cohort studies, or randomised controlled trials or non-randomised controlled trials; and (f) used all instruments/tools available to date in the field of UNC [23]. Therefore, this study excluded (a) descriptive studies, editorials, letters to the editor, qualitative studies, reviews, commentaries, books, chapters of books, books of congress, or presentations; (b) studies not addressing UNC data, not involving nurses/nursing aides, or not comparing data; and (c) studies not available in their full text. Inclusion and exclusion criteria are summarised in Supplementary Table 1.

### Search methods and study selection process

**Fig. 1:**
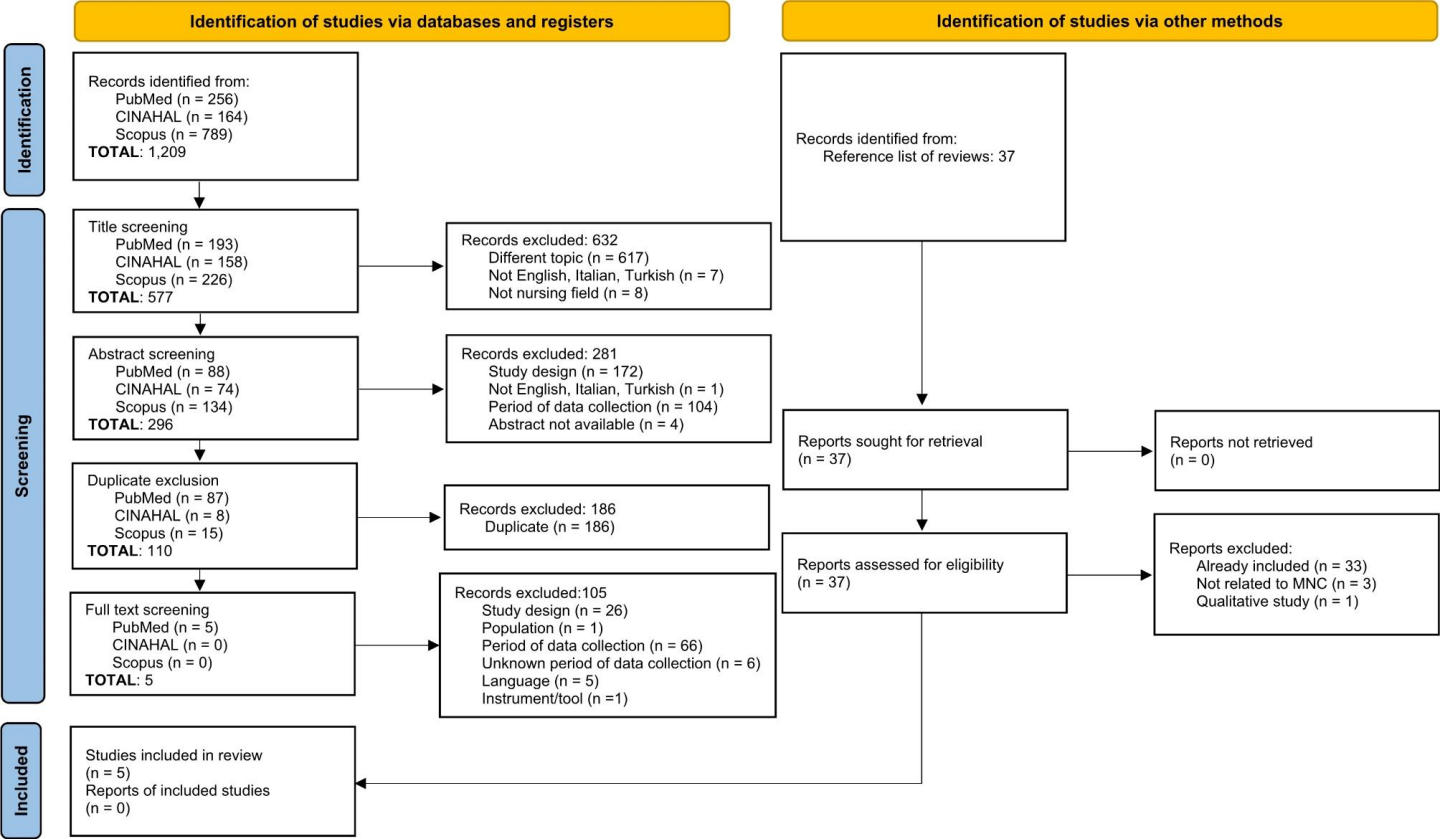
PRISMA 2020 flow diagram for new systematic reviews which included searches of databases, registers and other sources

Three electronic databases were searched using the keyword and search strings (Supplementary Table 2): Medline-PubMed, Cumulative Index to Nursing and Allied Health Literature (CINAHL), and Scopus. The reference lists of the retrieved studies were checked by two researchers (SC, AG) independently and then agreed upon. Three consequent screening stages were performed. At first, the titles of the retrieved studies were evaluated for their eligibility by two researchers (AB, SC); from 1,209 studies, we included 577 articles. Second, two researchers (AB, SC) screened the abstracts to evaluate eligibility. At this stage, 281 studies were excluded and 296 studies included for the next step. Moreover, duplicates were excluded (n = 186), and 110 studies remained. Finally, full texts of studies were evaluated for eligibility by two researchers (SC, AG), and studies excluded were categorised according to the reasons for exclusion (Fig. 1). Researchers performed the evaluation independently and then agreed upon it in all stages. Discrepancies were discussed with a third researcher (AP). In those cases where the data collection period was not declared, the corresponding author of the study was contacted; a total of 15 corresponding authors were contacted, and nine responded. We sent two gentle reminders to the remaining six corresponding authors. According to their missed reply, we decided to exclude these studies due to the uncertainty of the data collection period. At the end of the screening process, five studies were included (Fig. 1).

### Data extraction

Three reviewers (AB, SC, AG) extracted data using a grid developed and then piloted in two studies, where no changes were required. The grid was aimed at extracting the following data: (a) author(s), year, and country; (b) study design; (c) aim(s); (d) setting(s); (e) main characteristics of the participant nurses (and nursing aides) exposed to the COVID-19 patients and not, and the period of data collection for both; (f) data collection instrument and methods; and (g) UNC data on occurrence, reasons, and consequences. The extracted data were checked by the senior author (AP). Missed data (e.g., when the data were not reported in the publication) were managed by consulting the authors of the studies via email. In one case [24], according to the author of the study, the missed p-values were estimated (LG). By considering the available information reported in the study (conditional means and standard deviations), the test statistics were computed using the classical two samples mean comparison t-statistic formulation. Given the balanced sample sizes (n = 130 for each sample), pooled variance and different variance formulations are equivalent. Therefore, the p-values were computed considering a two-sided hypothesis testing framework (the reference distribution is a students’ t with 258 degrees of freedom).

### Quality assessment

The risk of bias was assessed using the Johanna Briggs Quality Appraisal Tools (e.g., analytical cross-sectional studies) [25]. Two reviewers (AB, SC) conducted the process independently. In case of disagreement, a third reviewer (AG) was consulted to reach a consensus. The quality evaluation of the study did not involve members of the research team if they were authors of an included study. At the evaluation, the included studies satisfied most of the quality appraisal criteria [25], except for items five (“Were confounding factors identified?”) and six (“Were strategies to deal with confounding factors stated?”), ranging from no [26] to unclear (in all studies), as summarised in Supplementary Table 3. Given that conducting a research process was challenging during the pandemic [27], the findings of the quality evaluation were not considered as exclusion criteria. To prevent bias, the following strategies were applied: (a) all researchers were involved in the study protocol refinement; (b) the literature search was conducted by two researchers independently; (c) the data extraction was performed by three researchers independently and supervised by the senior researcher; and (d) a meeting accompanied each stage, and the decision to move on to the next stage was made collectively.

### Synthesis

The included studies were synthesised using a narrative process to provide an integrated textual interpretation of the emerging results [28]. A preliminary synthesis was performed [28] to develop an initial description of the main characteristics of the studies. Then, the findings of the studies were systematically synthesised using a tabulation tool according to the research questions [28]. In the included studies, the MISSCARE Survey and the UNC Survey tools were used, articulated in Section A (= items measuring the elements of UNC, one-dimensionality scale) [23] and Section B (= items measuring the reasons for UNC, multidimensional scale) [23], thus providing the occurrence and the reasons at the overall, factor, or item level. Therefore, UNC occurrence and reasons were summarised in their statistical significance at the overall, factor, or item level according to the scale [23]. Data regarding the UNC consequences were also searched in the findings section of the manuscripts and summarised. All authors were involved in the data synthesis process: the work was divided and conducted independently and then in couples in order to ensure rigour and check the data.

## Results

### Study characteristics

Five studies were included (Fig. 1), and all are comparative in their design. As summarised in Table 1, two were published in 2021 [29, 30] and the remaining in 2022. Data collection periods ranged from March 2020 [24] to May 2021 [31] for the exposed group and from October 2019 [29] to January 2021 [26] for the comparison group.

**Table 1:** Main characteristics of the included studies according to the study pattern comparison

Author(s) Year Country Design	Aim(s)	Setting	Exposed *COVID-19 patients*	Comparison *Non-COVID-19 patients*	Instrument of data collection
**COVID-19 patients, first wave vs. before the pandemic in the same setting**					
Alfuqaha et al. [24] 2022 Jordan Comparative cross-sectional study	To compare the nurses’ perception of missed patient care before and during the COVID-19 To examine the relationship between MNC and other factors, such as job satisfaction, absence, and plans of leaving the current position	Medical/surgical wards and intensive care units Tertiary hospital in Amman	150 nurses, 130 answered (86.7%) Age: 25–34 years 94 (72.3%) Female: 84 (64.6%) Bachelor: 80 (61.5%) Nursing experience: 5 to 10 years, 50 (38.5%) Data collection: March-May 2020	150 nurses, 130 answered (86.7%) Age: 25–34 years 107 (82.3%) Female: 69 (53.1%) Bachelor: 92 (70.8%) Nursing experience: 5 to 10 years, 54 (41.5%) Data collection: November 2019 to January 2020	MISSCARE survey – Arabic version (based on Kalisch & Williams, 2009) Self-administered via paper and pencil
Nymark et al. [29] 2021 Sweden Cross-sectional study with a comparative approach	To evaluate MNC and patient safety during the outbreak and first wave of the COVID-19 pandemic in the inpatient wards	Cardiology department: two highly specialised medical wards and two intensive coronary care units Karolinska University Hospital in Stockholm	105 RNs and 140 NAs, 20 RNs and 23 NAs answered (19.0% and 16.4%) Age: median 36.7 years Female: 39 (90.7%) Bachelor: 18 (90.0%) Nursing experience: NR Data collection: May–June 2020	28 RNs and 31 NAs, participants’ rates NC Age: median 39.9 years Female: 50 (90.7%) Bachelor: 18 (69.2%) Nursing experience: NR Data collection: October 2019	MISSCARE survey – Swedish version (Nymark et al., 2020) Self-administered paper questionnaires
von Vogelsang et al. [30] 2021 Sweden Comparative cross-sectional study	To evaluate frequencies, types of, and reasons for MNC during the COVID- 19 pandemic in inpatient wards	Medical/surgical departments Karolinska University Hospital in Stockholm	235 RNs and 289 NAs, 130 RNs and NAs answered (24.8% total participants’ rates) Age: median 34.0 years Female: 112 (86.2%) Bachelor: 46 (78.0%) Nursing experience: > 10 years, 41 (31.5%) Data collection: May–June 2020	915 nursing staff (50% RNs), 248 answered (27.1%) Age: median 35.5 years Female: 126 (80.3%) Bachelor: 55 (69.6%) Nursing experience: > 10 years, 58 (36.9%) Data collection: October 2019	MISSCARE survey – Swedish version (Nymark et al., 2020) Self-administered paper questionnaires
**COVID-19 patients, second and third waves vs. before the pandemic in the same setting**					
Falk et al. [31] 2022 Sweden Comparative cross-sectional study	To describe reported MNC in the critical care context during different waves of the COVID-19 pandemic	Four critical care units (thoracic, neurosurgical, and two general critical care units) University Hospital in Stockholm	242 RNs during second wave, 38 answered (15.7%) 198 RNs during third wave, 37 answered (18.7%) *Second wave* Age: median 41.0 years Female: 34 (89.5%) Master or higher: 22 (57.9%) Nursing experience: > 10 years, 23 (60.5%) *Third wave* Age: median 50.0 Female: 32 (86.5%) Master or higher: 19 (51.4%) Nursing experience: > 10 years, 24 (64.9%) Data collection: November 2020 (second wave), May 2021 (third wave)	221 RNs, 59 answered (26.7%) Age: median 43.5 years Female: 49 (83.1%) Master or higher: 44 (74.6%) Nursing experience: > 10 years, 40 (67.8%) Data collection: October 2019	MISSCARE survey – Swedish version (Nymark et al., 2020) Self-administered online survey
**COVID-19 vs. non-COVID-19 patients, second wave**					
Cengia et al. [26] 2021 Italy Comparative cross-sectional study	To measure the occurrence of and reasons for UNC among COVID-19 and non-COVID-19 patients as perceived by nurses	22 units medical, geriatric, medical-surgical, and orthopedic units 15 COVID-19 and 7 non-COVID-19 units Two hospitals in Veneto region	479 RNs, 90 answered (72.8%*) Age: 40.1 (CI 37.8–42.3) years Female: 80 (88.9%) Bachelor: 49 (56.3%) Nursing experience: 16.1 (CI 13.7–18.6) years Data collection: November 2020-January 2021	479 RNs, 200 answered (72.8%*) Age: 37.7 (CI 36.2–39.2) years Female: 169 (84.5%) Bachelor: 137 (72.2%) Nursing experience: 13.5 (CI 11.9–15.1) years Data collection: November 2020 to January 2021	Unfinished Nursing Care Survey (Bassi et al., 2020) Self-administered online survey

Abbreviations: CI, confidence interval; COVID-19, coronavirus-19; MNC, missed nursing care; NA, nursing assistant; NC, not calculated; NR, not reported; RNs, registered nurses; UNC, Unfinished Nursing Care

* Total participation rate (exposed and comparison)

Three studies were conducted in Sweden [29–31], one in Jordan [24], and one in Italy [26]. Studies were aimed at comparing COVID-19 and non-COVID-19 patients by considering UNC data collected (a) before and in the first wave [24, 29, 30]; (b) before and in the second and third waves [31]; and (c) simultaneously during the second wave of the pandemic [26]. Only one study was multicentric [26], involving two healthcare trusts and 22 units; the remaining were mono-hospital based, involving medical and surgical [24, 26, 29, 30], intensive care [24, 29], and geriatric/orthopaedic units [26], whereas Falk et al. [31] included four critical care units.

Nurses included in the studies range from 37 [31] to 130 [24, 30] in the exposed group (caring for COVID-19 patients) and from 59 [29, 31] to 200 [26] in the comparison group (caring for non-COVID-19 patients). Samples involved nurses, while Nymark et al. [29] and von Vogelsang et al. [30] included registered nurses and nursing aides. Participation rates ranged from 15.7% [31] to 86.7% [24] in the exposed group and from 26.7% [31] to 86.7% [24] in the comparison group.

Nurses involved were mainly female in both the exposed (64.6% or above) and comparison groups (53.1% or above), with no statistical differences between groups. Nurses were slightly younger in the study by Alfuqaha et al. [24], where the majority in both groups (exposed 72.3% vs. comparison 82.3%) were aged 25–34 years, without significant differences. In the remaining studies, the average or median age was higher, without any statistical difference between the exposed (median age of 34 years or higher) and comparison groups (median age of 35.5 years or higher). In all studies, most nurses were educated at the bachelor level (exposed 56.3% or higher vs. comparison 69.2% or higher), with no statistical differences, except for the study by Cengia et al. [26], where the proportion of nurses educated at the bachelor levels was higher in the comparison group (72.2%) compared to the exposed group (56.3%) (p = 0.009). Moreover, most nurses in the study by Falk et al. [31] were educated at the master level or higher (exposed 57.9% and 51.4% in the second and third wave, respectively, vs. comparison 74.6%), without any difference across groups. This was also the study where the nursing experience was > 10 years for most nurses (exposed 47.4% and 48.6% in the second and third wave, respectively, vs. comparison 39.0%), without any difference across groups. On the other hand, the study by Alfuqaha et al. [24] reported nursing experience mainly between 5 and 10 years (exposed 38.5% vs. comparison 41.5%), without any statistical differences.

The MISSCARE Survey tool were used in all studies, except for the study by Cengia et al. [26], where the UNC Survey was adopted. Paper and pencil were mostly used [24, 29, 30], while two studies used an online survey with institutional emails to access nurses.

### Unfinished nursing care occurrence

At the global level, only one study [24] reported a statistical difference in the overall score including all MISSCARE Survey items (Supplementary Table 4), indicating a UNC occurrence perception slightly higher among nurses caring for COVID-19 patients compared to those caring for non-COVID-19 patients (3.37 [out of 5 “never missed”] vs. 3.68, p = < 0.001). At the items level, in nine (e.g., attending interdisciplinary care conferences), statistical differences emerged in studies using the MISSCARE Survey, including a total of 24 items; in the study using the UNC Survey, no statistical differences emerged in 36 out of 37 items (e.g., discussing with physicians and other staff members the problems and the interventions needed by patents) [26]. Some trends emerged in the remaining items at the study levek and across studies, as reported in Table 2.

**Table 2:** Significative differences in the Unfinished Nursing Care occurrence and reasons

Study patterns	COVID-19 patients, first wave vs. before the pandemic			COVID-19 patients, second and third waves vs. before the pandemic	COVID-19 vs. non-COVID-19 patients, second wave
SECTION A, interventions unfinished^§^ *Items and total scores*	Alfuqaha et al., 2022 [24]	Nymark et al., 2021 [29]	von Vogelsan et al., 2021 [30]	Falk et al., 2022 [31]	Cengia et al., 2021 [26]
Turning patient every two hours	↑	↑			
Ambulation three times per day or as ordered	↑	↑		↓ ↓	
Emotional support to patient and/or family	↑				
Mouth care			↓	↓ ↑	
IV/central line site care and assessments according to hospital policy	↑				
Skin/wound care	↑	↑			
Feeding patient when the food is still warm	↑				
Medications administered within 15/30 minutes before or after scheduled time		↓			↓
Assist with toileting needs within 5 min of request	↑			↓ ↓	
Focused reassessments according to patient condition	↑				
Response to call light is initiated within 5 min	↑	↑	↑		
Full documentation of all necessary data	↑				
Nursing staffs’ hand washing	↑				
Setting up meals for patients who feed themselves		↓	↓		
Monitoring intake/output	↑				
Bedside glucose monitoring as ordered	↑				
Vital signs assessed as ordered				↓ ↓	
*Total scores according to the tool*	↑				
**SECTION B, reasons** ^**§**^ *Items and total scores*					
Medications were not available when needed	↑			↓ ↓	
Supplies/equipment not available when needed	↑			↓ ↓	
Urgent patient situations (e.g., a patient’s condition worsening)	↑				
Inadequate number of assistive personnel (e.g., nursing assistants)					↑
Inadequate nursing care model (e.g., functional task-oriented model of care)					↑
Tension or communication breakdowns with other ancillary/support departments	↑				
Tension or communication breakdowns with the medical staff	↑				
Tension or communication breakdowns within the nursing team	↑				
Lack of backup support from team members	↑				
Inadequate hand-off from previous shift or sending unit	↑				
Nursing assistant did not communicate that care was not provided	↑				
Caregiver off unit or unavailable	↑				
*Factor: Communication*	↑				
*Factor: Material resources*	↑				
*Total scores in the tool*	↑				↑

Legend: § only those items/factors reporting statistically significant findings in the included studies are reported (see Supplementary Table 2)

↑ statistically higher among nurses caring for COVID-19 patients vs. the comparison; ↓ statistically lower among nurses caring for COVID-19 patients vs. the comparison. When no symbols are indicated, no statistical differences were reported by the included studies

Specifically, at the study level, Alfuqaha et al. [24] reported a significant difference in 14 items, all in the same direction, indicating that nurses caring for COVID-19 patients perceived a high occurrence of UNC. On the contrary, Cengia et al. [26] found only one statistical difference in “medication administration within 15/30 minutes before or after the scheduled time” less often missed among COVID-19 patients (2.32 [out of 5 “always unfinished”] vs. 2.72, p = 0.006). Similarly, Falk et al. [31] in the second and third waves reported significantly less UNC occurrence among nurses caring for COVID-19 patients in four items compared to the pre-pandemic, with the exception of “mouth care”, where the occurrence was less missed in the second (5.6%) and high in the third wave (27.0%) compared to the pre-pandemic data (23.7%). Mixed directions emerged instead in the remaining two studies, where less occurrence among COVID-19 patients was reported in two (e.g., “mouth care”, 30.4% missed vs. 48.4%, p = 0.003) out of three items [30] and statistically significant in two out of six (e.g., “medication administration within 15/30 minutes before/after the scheduled time” 11.5% vs. 34.8%, p = 0.050) [29].

Across studies (Table 2), significantly more UNC among COVID-19 patients was reported compared to non-COVID-19 patients in some items (e.g., “turning patient every two hours”, “providing skin/wound care”, “responding within 5 minutes”) (e.g., [24, 29, 30]). However, in other items, mixed findings emerged, in some higher UNC among COVID-19 patients (e.g., “ambulation three times per day”) [24, 29] and in others less [31]. Moreover, significantly less UNC among COVID-19 patients (“medication administered within 15/30 minutes before or after the scheduled time”) also emerged [26, 29].

### Unfinished nursing care reasons

At the overall level, two studies [24, 26] reported a statistical difference in the overall scores (Table 2, Supplementary Table 4), indicating a significantly higher contribution of reasons perceived by nurses caring for COVID-19 patients compared to those caring for non-COVID-19 patients (3.12 [out of 4 “significant reason”] vs. 2.90, p = < 0.001, and 2.21 vs. 2.07, p = 0.030, respectively). However, in five items (e.g., “inadequate nursing care model”), statistical differences emerged in studies using the MISSCARE Survey, ranking a total of 17 reasons, whereas no statistical differences emerged in 16 out of 18 items in the study using the UNC Survey (e.g., “inadequate number of staff”) [26]. Some trends emerged in the remaining UNC reasons at the study level and across studies, as reported in Table 2.

At the study level, Alfuqaha et al. [24] documented statistical differences in 12 items (e.g., “supplies were not available when needed” 3.17 [out of 4 “significant reason”] vs 2.85, p = 0.002) and in two factors (“communication” [3.02 vs. 2.74, p < 0.001], “material resources” [3.19 vs. 2.85, p < 0.001]). However, all differences are in the same direction, indicating that nurses caring for COVID-19 patients perceived these as important reasons triggering UNC. Differently, Cengia et al. [26] found only two statistical differences in “inadequate number of nursing aides” (1.88 vs. 1.58, p = 0.003) and “inadequate nursing care model” (2.79 vs. 2.50, p = 0.016), which emerged as significant reasons triggering UNC among COVID-19 patients compared to non-COVID-19 patients.

Falk et al. [31] instead reported a significantly inferior contribution in triggering UNC among COVID-19 patients compared to non-COVID-19 patients in “medication was not available when needed” (26.3% [second wave], 18.9% [third wave] vs. 43.1% [pre-pandemic], p = 0.003) and in “supplies and equipment were not available when needed” (5.3% vs 2.9% vs 26.3%, p = 0.001). Across studies (Table 2), contrasting findings have been reported between two studies, the first [24] indicating a significantly higher contribution of two items in triggering UNC among COVID-19 patients and the second indicating a significantly lower contribution of the same items among COVID-19 patients [31].

### Unfinished nursing care consequences

No studies documented the consequences of UNC on patients.

## Discussion

### Methodological issues

We included studies published since 2020 by carefully inspecting the data collection period considered by each: COVID-19 cases and deaths have been reported since December 2019 in some countries, while the formal announcement of the pandemic occurred on 11 March 2020 [20]. However, the health service capacity was expanded at the beginning of the escalation phase [32] and this may have also affected nursing care [33]. With the accumulation of evidence clarifying the whole history of the pandemic, updating this review regarding the right period of inclusion might be important to describe its effects on UNC over the waves comprehensively, as the continuing restructuring of the health service, on the one hand, and the fatigue of nurses, on the other, may have also influenced the perceptions.

We decided to include only comparative studies aimed at confronting data in the same settings to minimise the role of other factors on UNC data documented at the unit levels [9]. This decision ensures increased homogeneity of the external factors affecting nurses during the care of both groups of patients, whereas it should be acknowledged that some additional internal factors (e.g., psychological stress) generated by the COVID-19 pandemic may have influenced the perception of nurses involved in the pandemic period, as well as across waves, compared to those involved in the pre-pandemic period. Summarising all studies performed during the pandemic without comparative intents may inform on the methodological challenges encountered in doing research during a pandemic and on UNC occurrence and reasons in challenging times when care routines are disrupted [34]. Having a comprehensive baseline may also constitute a new reference point for the post-pandemic studies in the field of UNC, given that the healthcare settings in the pre-pandemic period were characterised by different priorities, work conditions, and issues, threatening the comparison [35].

Some missed data of the studies were collected from authors or estimated by the research team. We decided to adopt a co-constructive approach by involving authors, given the difficulties in doing research during the pandemic [27] that may have affected its quality. However, the quality of the included studies, as evaluated with the Joanna Briggs Institute [25] checklist, was appropriate in all items, except for those regarding the confounding factors that have been less precisely described. The work environments and the nursing care processes were chaotic (e.g., [36]); thus, taking into account all confounding factors was a challenge. Moreover, while the exposed group (COVID-19 patients) was substantially homogeneous, the comparison group (e.g., medical surgical, critical care patients) may have been affected by different health issues requiring different elements of care. Imbalances in the main profile of patients may have introduced biases in the nurses’ perceptions.

### Study characteristics

A few UNC comparative studies have been performed in different countries. In the attempt to establish differences, if any, three main patterns of comparison have emerged: (1) UNC before and during the pandemic (first wave), (2) before and during different waves of the pandemic (second and third), and (3) simultaneously during the pandemic (second wave) comparing COVID-19 and COVID-19-free units. The first and second patterns were prevalent given that four studies based their comparison on data collected before the pandemic (e.g., October 2019 [29]) and then involved the same settings to detect changes: in these cases, a well-established line of research (e.g., [29]) may have allowed the rapid development of the research action by repeating the same protocol used at the baseline and across different waves (e.g., [31]). Differently, only one study [26] compared at the same time the perceptions of nurses caring for COVID-19 patients and of those caring for patients in COVID-19-free units. It was likely difficult to conduct simultaneous comparisons, not only for the extremely critical conditions of the nursing staff where the priority was to prevent any form of additional burden (e.g., filling in a questionnaire), but also because of the continuous adaptation of the unit’s missions, with some of them exclusively dedicated to COVID-19 patients [26].

One study collected data early, in March 2020 [24], thus describing the first wave and suggesting the capacity of researchers to be ready to detect an emerging issue. One collected data mirroring a longitudinal approach, with repetitive data collections considering the second and third waves [31], while one used a simultaneous approach by collecting data in the second wave [26]. Data were collected up to May 2021 for the exposed and in 2019 or 2021 for the comparison group: as a consequence, the available findings reflect substantially the first year of the pandemic, when some strategies to increase the service capacity were implemented. Therefore, long-term consequences of these changes have not been evaluated [37]. Some studies may have been performed later and/or submitted to a journal for peer review. In the initial stage of the pandemic, the reviewing process of journals was speedy, but it became slower in recent times because of the increased number of manuscripts submitted and the progressive “normalisation” of the pandemic [38], rendering studies in the field less important. Moreover, studies were mainly monocentric in nature and conducted in hospitals [39]: medical, surgical, and critical care settings were considered, reflecting the main settings already included in this field of research [23]. Data regarding UNC in the community or residential settings have not been provided, likely due to the lack of instruments measuring the issues in these settings [23].

The sample size was limited mainly in the exposed groups of nurses, with a variable participant rate, from very low (15.7%) to high (86.7%), suggesting difficulties in involving nurses during the pandemic that may be due to the dramatic times experienced by them and the prevailing clinical priorities. In addition, the methods of data collection based mainly on paper and pencil (e.g., [24]) may have prevented participation due to the fear of being infected by touching a surface contaminated with the virus [40]. Safety reasons and green healthcare research approaches suggest improving the use of online surveys.

The profile of participant nurses is in line with that documented in previous studies in the field (e.g., [16]), where mainly females, experts, and those educated at the bachelor level were involved. Most of the individual characteristics were homogenous between the exposed and the comparison nurses, except for education in the study by Cengia et al. [26], where fewer nurses with a bachelor’s degree were in the exposed group compared to the comparison group. Individual variables have been underlined as affecting the perception of UNC among nurses; therefore, their homogeneity between the groups may have prevented confounding factors. In the case of education, higher levels have been documented to increase UNC [9], but more studies are needed to increase the understanding of how nurses set priorities while coping with complex conditions.

Data collected with the MISSCARE Survey tools were used in all studies, allowing comparison of the findings. Cengia et al. [26] used the UNC Survey that was developed from the MISSCARE Survey, thus sharing the main items [41] in both Section A, measuring the elements of unfinished care, and Section B, measuring the underlying reasons as perceived by nurses.

### Unfinished nursing care occurrence

Interesting trends emerged in the UNC occurrence that can be interpreted under three main lines. First, the overall score was significantly different at the study level in only one study [24], where several items, all in the same direction, indicated a higher UNC occurrence among COVID-19 patients. The study was performed in the first wave when nurses were unprepared, exposed to unprecedented organisational changes, emotionally burdened, fearful, and in high levels of uncertainty [42]. Conversely, no differences, except for one item indicating less UNC among COVID-19 patients, were found by Cengia et al. [26] in their study conducted in the second wave. Similarly, Falk et al. [31] mainly found less UNC among COVID-19 patients in the second and third waves. After the first wave, nurses were more prepared, and healthcare services were prioritised towards COVID-19 units, putting non-COVID-19 patients at risk to receive less care [43]. In those studies [29, 30] reporting mixed directions (in some items increased and in others decreased UNC among COVID-19 patients), this may be related to the different settings of care (intensive care vs. medical and surgical units) but also to the prioritisation of different needs (e.g., mouth care, administering medications on time for COVID-19 patients) that may have changed the process of care.

At the item level, less care was given to COVID-19 patients across studies in “turning patients every two hours”, “providing skin/wound care”, and “responding to call light within 5 minutes”, while increased care was given in “setting up meals for patients who feed themselves” and “medication administration within 15/30 minutes of the scheduled time”. Care was mixed in other items (“ambulation three times a day”, “mouth care”, “assisting with toilet needs within 5 minutes of request”). Researchers have documented similarities in the elements of unfinished care and in their order, suggesting a stable pattern across countries and over the years [44]. This order seems to be altered during the pandemic. The model of care delivered in the units assisting COVID-19 patients was based mainly on intervention clusterisation (*“I enter the room and take care of all the patient*’*s needs”*) to avoid cross-contamination and decrease the fatigue level related to the use of PPE [45], and this may have changed the patterns of care (e.g., “being able to respond rapidly to a call light when I assist another patient”). Moreover, the context of care was changed, limiting the spread of the virus, and this may explain why in some studies “ambulating the patients three time a day” was missed [24, 29] while in others it was ensured [31], possibly due to the care setting allowing or limiting movements. Surprisingly, relational care (e.g., “emotional support”) was more significantly unfinished among COVID-19 patients only in one study [24] conducted in the first wave and not in the others; this may indicate the effects of strategies used by nurses to compensate for the potential lack [46, 47] as well as a sort of “normalisation” in both groups regarding the limited emotional care provided due to the PPE, family visiting restrictions, and the limited personal contact recommended to prevent virus diffusion.

Some interpretations of the findings may also be offered at the instrument level measuring UNC. For instance, COVID-19 patients were recommended to be kept in the prone position [48]. Thus, the item “turning patients every two hours” mainly to prevent pressure sores may have been misinterpreted or attributed less meaning. Moreover, the list of items included was not able to reflect the practices required to prevent the spread of infection as infection prevention/control programmes [49]. Thus, instruments may have missed the capacity to capture some specific elements of care required during the pandemic.

### Unfinished nursing care reasons

First, all reasons were different between COVID-19 and non-COVID-19 patients at the overall level only in two studies [24, 26], but in the item analysis, the specific factors triggering UNC were opposite: several and composite in the first wave [24], suggesting that the units were unprepared to support nursing care, and limited and specific (regarding nursing aides and nursing care models) in the second wave [26]. Although studies were conducted in different countries, this may suggest that facilities increased their support for COVID-19 units and nursing care over the waves. This seems to be true also in the case of two specific items - “medications were not available when needed” and “supplies and equipment were not available when needed” - which were significant reasons in the first wave [24] but not in the second and third waves [31]. This may confirm that in the initial phases of the pandemic, the resources were lacking and then prioritised and provided to COVID-19 patients. Statistical differences emerged when comparing the pre-pandemic period and the following waves [31], suggesting that resources were devoted mainly to COVID-19 patients.

Two studies did not find any differences in the reasons [29, 30], which may be interpreted at the instrument level, as the instruments may have been unable to capture all factors triggering UNC, given that they were developed before the pandemic. For example, the lack of experience among nurses deployed from one unit to another to increase the care capacity [15] is not contemplated among the reasons for UNC, which instead reflects the routine deficiencies and not those in extraordinary circumstances like during the pandemic. The development of tools measuring UNC reasons in specific situations may be recommended for future studies.

### Unfinished nursing care consequences

No UNC consequences have been documented to date suggesting and area of further investigation. The limited time available and the critical conditions of the healthcare services [50], where follow-ups have been interrupted, may have prevented any attempt.

### Limitations

This review is affected by several limitations. Six studies were excluded from the inclusion process because no answer was obtained despite attempts to contact the authors to assess when data were collected. We included studies comparing UNC data collected during the pandemic with data collected before: in all these studies [24, 29–31], the pre-pandemic period ranged from October to November 2019, just before the outbreak. Other excluded studies (e.g., [14]) could have had data from the pre-pandemic period available, and they could have been asked for the database to evaluate the differences, if any. However, according to the aims of the review, we decided not to perform any secondary analysis. Furthermore, while assessing the reasons for UNC, only those collected with validated instruments were considered without considering additional variables explored in the included studies at the different levels (e.g., nurse-to-patient ratio) due to the variability of data collected across studies preventing any form of comparison and summarisation of the findings to detect trends, which was the main intent of this review.

### Relevance to clinical practice

UNC is universally used as an indicator of the quality of nursing care. Findings indicate that the first wave, that of the escalation in which hospitals urgently expanded their capacity to ensure care for COVID-19 patients, was the wave in which nurses perceived higher UNC among these patients. In later stages, non-COVID-19 patients were more exposed to UNC. A similar trend also emerged among the reasons, suggesting that the prioritisation of the system towards COVID-19 patients caused missed care of non-COVID-19 patients by reducing resources. This suggests that nurses need support in transiting and adapting the models of care in the escalation phase of healthcare services while dealing with a pandemic, and later those nurses caring for other patients should be supported in providing the expected care. Furthermore, with respect to UNC reasons, the long-term effects on the nurses of both cohorts generated by the turbulent organisational conditions and the nursing care routine disruptions can cause increased fatigue and UNC, suggesting the need for additional support also when the pandemic is over. Moreover, because decisions regarding UNC minimisation must be timely, a standardised electronic tracking system assessing UNC occurrence in the e-health record systems is suggested. Raising awareness among both clinical nurses and managers might help them to recall the need for interventions and might also inform decisions regarding the supports/resources needed. Additionally, developing care protocols facilitating the decision-making processes of nurses against the unknown, especially when deployed from one unit to another, without appropriate training, might be helpful. Conducting a continuous internal evaluation for UNC would minimise its occurrence: in this context, a systematic intentional rounding intervention can be useful.

Above all, it is important to continue to investigate UNC during the pandemic, especially in this phase in which the return to the normality allows researchers to proceed with more accuracy in their investigations. Many studies could be carried out as secondary analysis if researchers who collected data during the pandemic (even in the waves following the second to which the included studies refer) compared with those collected before, in the same settings, are available. Furthermore, studies investigating long-term effects of UNC would be needed to allow a fuller understanding of the phenomenon among both COVID-19 and non-COVID-19 patients.

## Conclusion

To the best of our knowledge, this is the first systematic review summarising UNC between two populations, namely COVID-19 and non-COVID-19 patients, as perceived by nurses in the dramatic conditions experienced during the pandemic. The COVID-19 pandemic establishes different priorities for the system and for nursing care delivery, which can be highlighted by analysing UNC. Knowing differences, if any, between COVID-19 and non-COVID-19 patients as perceived by nurses offers a reflection on the quality of nursing care provided in difficult times, also with regards to non-COVID-19 patients who were less prioritised by the system and also by the researchers.

A few studies have been published in this field. From a methodological point of view, the five retrieved reflect three main profiles of comparison of the data collected: (a) before the pandemic and in the first wave; (b) before and in the second and third waves; and (c) simultaneously during the second wave of the pandemic. Regarding UNC occurrence, three patterns emerged, indicating a higher occurrence among COVID-19 patients in the first wave, less occurrence among them compared to non-COVID-19 patients in the second wave, and mixed findings, with some in favour and others in contrast to COVID-19 patients. Similar patterns also emerged with regard to UNC reasons, with a significant role of the lack of material resources in the first wave among COVID-19 patients, while in the second and third waves these reasons became more important for non-COVID-19 patients. However, there are no data on the consequences for patients.

### Electronic supplementary material

Below is the link to the electronic supplementary material.

Supplementary Material 1

Supplementary Material 2

Supplementary Material 3

Supplementary Material 4

## Data Availability

Not applicable.

## List of abbreviations

CINAHL: Cumulative Index to Nursing and Allied Health Literature
COVID: Coronavirus
PECO: Population, Exposure, Comparator, and Outcomes
PPE: Personal Protective Equipment
PRISMA: Preferred Reporting Items for Systematic Reviews and Meta-Analyses
UNC: Unfinished Nursing Care
