# Supplementary material for: Were the unfinished nursing care occurrence, reasons, and consequences different between COVID-19 and non-COVID-19 patients? A systematic review
Source: BMC Nurs. 2023 Sep 27;22:341. doi: 10.1186/s12912-023-01513-4 (PMC10523650; doi:10.1186/s12912-023-01513-4)
Supplement: Supplementary file 1 — Supplementary Material 1 [file 12912_2023_1513_MOESM1_ESM.docx]

**Supplementary Table 4.** Significative differences in Unfinished Nursing Care occurrence and reasons according to the study patterns

| **Study patterns** | **COVID-19 patients first wave**  **vs before the pandemic** | | | **COVID-19 patients second and third wave**  **vs before the pandemic** | **COVID-19**  **vs non-COVID-19 patients, second wave** |
| --- | --- | --- | --- | --- | --- |
| **SECTION A, Elements of UNC missed ^§^** | **Alfuqaha et al., 2022^a^** | **Nymark et al., 2021^b^** | **von Vogelsan et al., 2021^b^** | **Falk et al., 2022^b^** | **Cengia et al., 2021^c^** |
| Turning patient every 2 hours | 2.97 vs 3.28 (p=0.019) | 76.3% vs 43.9% (p=0.003) |  |  |  |
| Ambulation 3 times per day or as ordered | 2.85 vs 3.23 (p=0.006) | 65.0% vs 39.7% (p=0.023) |  | 68.4% vs 59.5% vs 88.1% (p=0.004) |  |
| Emotional support to patient and/or family | 3.09 vs 3.42 (p=0.017) |  |  |  |  |
| Mouth care |  |  | 30.4% vs 48.4% (p=0.003) | 5.6% vs 27.0% vs 23.7% (p=0.040) |  |
| IV/central line site care and assessments according to hospital policy | 3.62 vs 4.02 (p=0.001) |  |  |  |  |
| Skin/Wound care | 3.52 vs 3.79 (p=0.050) | 43.2% vs 19.6% (p=0.020) |  |  |  |
| Feeding patient when the food is still warm | 3.19 vs 3.68 (p<0.001) |  |  |  |  |
| Medications administered within 15/30 minutes before or after scheduled time |  | 11.5% vs 34.8% (p=0.050) |  |  | 2.32 vs 2.72 (p=0.006) |
| Assist with toileting needs within 5 minutes of request | 3.30 vs 3.63 (p=0.014) |  |  | 40.5% vs 29.7% vs 56.6% (p=0.036) |  |
| Focused reassessments according to patient condition | 3.20 vs 3.67 (p<0.001) |  |  |  |  |
| Response to call light is initiated within 5 minutes | 3.42 vs 3.71 (p=0.041) | 18.4% vs 1.8% (p=0.006) | 15.4% vs 6.0% (p=0.015) |  |  |
| Full documentation of all necessary data | 3.48 vs 3.97 (p<0.001) |  |  |  |  |
| Nursing staffs’ hand washing | 3.55 vs 3.97 (p=0.005) |  |  |  |  |
| Setting up meals for patients who feed themselves |  | 2.5% vs 21.4% (p=0.007) | 7.1% vs 23.8% (p<0.001) |  |  |
| Monitoring intake/output | 3.68 vs 4.09 (p=0.003) |  |  |  |  |
| Bedside glucose monitoring as ordered | 3.78 vs 4.09 (p=0.024) |  |  |  |  |
| Vital signs assessed as ordered |  |  |  | 2.6% vs 0.0% vs 16.1% (p=0.007) |  |
| *Overall score* | 3.37 vs 3.68 (p<0.001) |  |  |  |  |
| **SECTION B, reasons**^§§^ |  |  |  |  |  |
| Medications were not available when needed | 3.28 vs 2.80 (p<0.001) |  |  | 26.3% vs 18.9% vs 43.1% (p=0.033) |  |
| Supplies/equipment not available when needed | 3.17 vs 2.85 (p=0.002) |  |  | 5.3% vs 2.9% vs 26.3% (p=0.001) |  |
| Urgent patient situations (e.g., a patient’s condition worsening) | 3.25 vs 3.02 (p=0.020) |  |  |  |  |
| Inadequate number of assistive personnel (e.g. nursing assistants, techs etc.) |  |  |  |  | 1.88 vs 1.58 (p=0.003) |
| Inadequate nursing care model (e.g., functional task-oriented model of care) |  |  |  |  | 2.79 vs 2.50 (p=0.016) |
| Tension or communication breakdowns with other ancillary/support departments | 3.07 vs 2.82 (p=0.013) |  |  |  |  |
| Tension or communication breakdowns with the medical staff | 3.05 vs 2.79 (p=0.009) |  |  |  |  |
| Tension or communication breakdowns within the nursing team | 3.04 vs 2.70 (p=0.001) |  |  |  |  |
| Lack of backup support from team members | 3.05 vs 2.76 (p=0.005) |  |  |  |  |
| Inadequate hand-off from previous shift or sending unit | 3.16 vs 2.71 (p<0.001) |  |  |  |  |
| Nursing assistant did not communicate that care was not provided | 2.85 vs 2.60 (p=0.015) |  |  |  |  |
| Caregiver off unit or unavailable | 2.98 vs 2.58 (p<0.001) |  |  |  |  |
| *Factor: Communication* | 3.02 vs 2.74 (p<0.001) |  |  |  |  |
| *Factor: Material resources* | 3.19 vs 2.85 (p<0.001) |  |  |  |  |
| *Overall score* | 3.12 vs 2.90 (p<0.001) |  |  |  | 2.21 vs 2.07 (p=0.030) |

^a^ Section A: 5-point Likert scale ranging from 5 “Never missed” to 1 “Always missed”. Section B: 4-point Likert scale ranging from 4 “Significant reason” to 1 “Not a reason for missed care”.

^b^ Section A: the Likert scale was dichotomised by considering ‘occasionally’, ‘frequently’ and ‘always’ as missed vs ‘rarely’ and ‘never’ as non-missed. Section B: the Likert scale was dichotomised by considering ‘significant’ and ‘moderate’ as reasons vs ‘minor’ and ‘not a reasons’ for missed care.

^c^ Part A: 5-point Likert scale ranging from 1 “never” to 5 “always unfinished”. Part B: 5-point Likert scale ranging from 5 “A very significant reason” to 1 “Not a significant reason”.

^§^ Any difference in the MISSCARE Survey items (Alfuqaha et al., 2022; Falk et al., 2022; Nymark et al., 2021; von Vogelsan et al., 2021): Attend interdisciplinary care conference whenever held; Assess effectiveness of medications; Patient discharge planning and teaching; Patient teaching about procedures, tests, and other diagnostic studies; Patient bathing/skin care; medication as needed requests acted on within 15 minutes; Patient assessments performed each shift.

Any difference in the following Unfinished Nursing Care items (Cengia et al., 2021): Perform bedside glucose monitoring as prescribed; Perform clinical handover to adequately inform the next shift nursing team about patients’ conditions; Record vital signs as planned; Provide personal hygiene to patients who need it; Monitoring intake/output; Prevent healthcare associated infections adopting good clinical practice (e.g., hand hygiene between patients); Monitor pain as planned; Collect data on the situation of the patients’ care at the beginning of the shift, through the handover; Ensure intensive surveillance, re-evaluating, those patients who are unstable or who present a risk of deteriorating conditions; Check pressure ulcers and change dressing according to protocols; Provide clinical teaching to nursing student; Prevent negative outcomes for patients at risk (e.g., falls, pressure ulcers, and malnutrition); Perform physical assessment (e.g., skin integrity and invasive device insertion site); Administer medications as needed within 15 min from the patient’s request; Monitor administered medications effects; Helping patients who are unable to eat independently and/or have clinical problems (e.g., dysphagia); Go to the patients at the bedside without being called; Fill in/update the clinical documentation/care plan in a comprehensive way; Helping patients who are unable to drink independently and/or have clinical problems; Teach patients and carers how to self-care at home; Document properly the interventions provided and revise the care plan; Ensure patients’ comfort (microclimate, patient positioning); Assess the effectiveness of the care provided, for example, reviewing if nursing care needs have been met; Involve patients and carers in the discharge planning; To stimulate the patient to maintain/improve his/her independence; Inform patients and their caregivers regarding the nursing care they are receiving; Respond promptly to patients’ calls (within 5 min); Supervise the tasks assigned to the nurse aides; Perform a round at the beginning of the shift to know the patients, present themselves, and deepen their situation; Emotionally support patients and carers by listening to their needs/concerns; Communicate with patients and carers; Discuss with physicians and other staff members the problems and interventions needed by patients; Help patients who need it to get in the chair; Spend time with patients and their carers; Provide mouth care to patients who need it; Passive mobilization/changing position in bedrest patient; Help patients in need in ambulation.

^§§^ Any difference in the MISSCARE survey reasons (Alfuqaha et al., 2022; Falk et al., 2022; Nymark et al., 2021; von Vogelsan et al., 2021). At the factor level: Labour Resources. At the item level: Unexpected rise in patient volume and/or acuity on the unit; Inadequate number of assistive and/or clerical personnel (e.g., nursing assistants, techs, unit secretaries, etc.); Inadequate number of staff; Unbalanced patient assignments; Heavy admission and discharge activity; Supplies/equipment not functioning properly when needed; Other departments did not provide the care needed.

Any difference in the following Unfinished Nursing Care reasons (Cengia et al., 2021). At the factor level: Communication, Priority setting; Nurses’ aides’ supervision; Material resources; Human resources, Workflow predictability. At the item level: Tension/conflicts within the nursing staff; Incomplete or interrupted communication among nursing staff; Tension/conflicts between nursing and medical staff; Incomplete or interrupted communication between nursing and medical staff; Lack of support/collaboration among team members; Inaccurate initial priority setting; Inadequate priority reassessment during the shift; Nurse aides missed or delayed reporting the tasks left undone; Incomplete or interrupted communication between nursing staff and nurse aides/assistive personnel; Other departments did not provide the service expected (e.g., delay in diagnostic processes); Medications prescribed not available; Equipment not available/not functioning properly when needed; Inadequate number of nurses; Inadequate number of nurse aides; Unexpected rise in patient acuity; Heavy admission/discharge activity during the shift.
