# Supplementary material for: Were the unfinished nursing care occurrence, reasons, and consequences different between COVID-19 and non-COVID-19 patients? A systematic review
Source: BMC Nurs. 2023 Sep 27;22:341. doi: 10.1186/s12912-023-01513-4 (PMC10523650; doi:10.1186/s12912-023-01513-4)
Supplement: Supplementary file 2 — Supplementary Material 2 [file 12912_2023_1513_MOESM2_ESM.docx]

**Supplementary Table 3.** Quality appraisal of included studies with Joanna Briggs Institute tool for analytical cross-sectional studies (Joanna Briggs Institute, 2020)

|  | **Alfuqaha et al., 2022** | | | **Cengia et al., 2021** | | | **Falk et al., 2022** | | | **Nymark et al., 2021** | | | **von Vogelsan et al., 2021** | | |
| --- | --- | --- | --- | --- | --- | --- | --- | --- | --- | --- | --- | --- | --- | --- | --- |
|  | Rater 1 | Rater 2 | **Consensus** | Rater 1 | Rater 2 | **Consensus** | Rater 1 | Rater 2 | **Consensus** | Rater 1 | Rater 2 | **Consensus** | Rater 1 | Rater 2 | **Consensus** |
| **Item 1**. Were the criteria for inclusion in the sample clearly defined? | Y | Y | **Y** | U | Y | **Y** | U | Y | **Y** | N | N-U | **U** | U | Y | **Y** |
| **Item 2**. Were the study subjects and the setting described in detail? | Y | Y | **Y** | Y | Y | **Y** | Y | Y | **Y** | Y | Y | **Y** | Y | Y | **Y** |
| **Item 3**. Was the exposure measured in a valid and reliable way? | Y | Y | **Y** | Y | Y | **Y** | Y | Y | **Y** | Y | Y | **Y** | Y | Y | **Y** |
| **Item 4**. Were objective, standard criteria used for measurement of the condition? | NA | Y | **NA** | NA | Y | **NA** | NA | Y | **NA** | NA | N-Y | **NA** | NA | Y | **NA** |
| **Item 5**. Were confounding factors identified? | Y | Y-U | **Y** | N | N | **N** | N | Y-U | **Y** | N | Y-U | **Y** | Y | N | **Y** |
| **Item 6**. Were strategies to deal with confounding factors stated? | Y | Y-U | **U** | N | N | **N** | N | Y-U | **U** | N | Y-U | **U** | Y | N | **U** |
| **Item 7**. Were the outcomes measured in a valid and reliable way? | Y | Y | **Y** | Y | Y | **Y** | U | Y | **Y** | Y | Y | **Y** | Y | Y | **Y** |
| **Item 8**. Was appropriate statistical analysis used? | Y | Y | **Y** | Y | Y | **Y** | Y | Y | **Y** | Y | Y | **Y** | Y | Y | **Y** |

**Abbreviations:** N, No; NA, Not Available; U, Unclear; Y, Yes.

Joanna Briggs Institute. (2020). *Checklist for analytical cross-sectional studies*. Retrieved in: https://jbi.global/critical-appraisal-tools
