# Supplementary material for: Were the unfinished nursing care occurrence, reasons, and consequences different between COVID-19 and non-COVID-19 patients? A systematic review
Source: BMC Nurs. 2023 Sep 27;22:341. doi: 10.1186/s12912-023-01513-4 (PMC10523650; doi:10.1186/s12912-023-01513-4)
Supplement: Supplementary file 3 — Supplementary Material 3 [file 12912_2023_1513_MOESM3_ESM.docx]

**Supplementary Table 2.** Keywords and search strings

| **PUBMED** | | | | | | | | | | | | | | | |
| --- | --- | --- | --- | --- | --- | --- | --- | --- | --- | --- | --- | --- | --- | --- | --- |
| **Search number** | **Query** | | |  | | | | **Filters** | | | **Search Details** | | | **Results** | **Time** |
| 3 | 1 AND 2 | | |  | | | | Time: 2020-2023 | | | ("nurse"[Title/Abstract] OR "nursing"[Title/Abstract]) AND ("missed care"[Title/Abstract] OR "missed nursing care"[Title/Abstract] OR "unfinished nursing care"[Title/Abstract] OR "unfinished care"[Title/Abstract] OR "implicit rationing of nursing care"[Title/Abstract] OR "implicit rationing"[Title/Abstract] OR "rationing of nursing care"[Title/Abstract] OR "rationed care"[Title/Abstract] OR "prioritization process"[Title/Abstract] OR "omitted nursing care"[Title/Abstract] OR "task left undone"[Title/Abstract] OR "task undone"[Title/Abstract]) | | | 256 | 01/01/2023  09:24 |
| 2 | (nurse[Title/Abstract]) OR (nursing[Title/Abstract]) | | |  | | | | Time: 2020-2023 | | | "nurse"[Title/Abstract] OR "nursing"[Title/Abstract] | | | 56,691 | 01/01/2023  09:23 |
| 1 | (((((((((((missed care[Title/Abstract]) OR (missed nursing care[Title/Abstract])) OR (unfinished nursing care[Title/Abstract])) OR (unfinished care[Title/Abstract])) OR (implicit rationing of nursing care[Title/Abstract])) OR (implicit rationing[Title/Abstract])) OR (rationing of nursing care[Title/Abstract])) OR (rationed care[Title/Abstract])) OR (prioritization process[Title/Abstract])) OR (omitted nursing care[Title/Abstract])) OR (task left undone[Title/Abstract])) OR (task undone[Title/Abstract]) | | |  | | | | Time: 2020-2023 | | | "missed care"[Title/Abstract] OR "missed nursing care"[Title/Abstract] OR "unfinished nursing care"[Title/Abstract] OR "unfinished care"[Title/Abstract] OR "implicit rationing of nursing care"[Title/Abstract] OR "implicit rationing"[Title/Abstract] OR "rationing of nursing care"[Title/Abstract] OR "rationed care"[Title/Abstract] OR "prioritization process"[Title/Abstract] OR "omitted nursing care"[Title/Abstract] OR "task left undone"[Title/Abstract] OR "task undone"[Title/Abstract] | | | 323 | 01/01/2023  09:22 |
| **CINAHL** | | | | | | | | | | | | | | | |
| **Search number** | | | **Query** | |  | | **Filters** | | | **Search Details** | | | | **Results** | **Time** |
| 3 | | | S1 AND S2 | |  | | Time: 2020-2023 | | | ("nurse"[Title/Abstract] OR "nursing"[Title/Abstract]) AND ("missed care"[Title/Abstract] OR "missed nursing care"[Title/Abstract] OR "unfinished nursing care"[Title/Abstract] OR "unfinished care"[Title/Abstract] OR "implicit rationing of nursing care"[Title/Abstract] OR "implicit rationing"[Title/Abstract] OR "rationing of nursing care"[Title/Abstract] OR "rationed care"[Title/Abstract] OR "prioritization process"[Title/Abstract] OR "omitted nursing care"[Title/Abstract] OR "task left undone"[Title/Abstract] OR "task undone"[Title/Abstract]) | | | | 164 | 01/01/2023  09:24 |
| 2 | | | TI nurse OR TI nursing | |  | | Time: 2020-2023 | | | "nurse"[Title/Abstract] OR "nursing"[Title/Abstract] | | | | 35,620 | 01/01/2023  09:29 |
| 1 | | | TI missed nursing care OR TI missed care OR TI unfinished nursing care OR TI unfinished care OR TI implicit rationing of nursing care OR TI implicit rationing OR TI rationing of nursing care OR TI rationed care OR TI prioritization process OR TI omitted nursing care OR TI task left undone OR TI task undone | |  | | Time: 2020-2023 | | | "missed care"[Title/Abstract] OR "missed nursing care"[Title/Abstract] OR "unfinished nursing care"[Title/Abstract] OR "unfinished care"[Title/Abstract] OR "implicit rationing of nursing care"[Title/Abstract] OR "implicit rationing"[Title/Abstract] OR "rationing of nursing care"[Title/Abstract] OR "rationed care"[Title/Abstract] OR "prioritization process"[Title/Abstract] OR "omitted nursing care"[Title/Abstract] OR "task left undone"[Title/Abstract] OR "task undone"[Title/Abstract] | | | | 229 | 01/01/2023  09:28 |
| **SCOPUS** | | | | | | | | | | | | | | | |
| **Search number** | | **Query** | | | |  | | | **Filters** | | | **Search Details** | **Results** | | **Time** |
| 3 | | 1 AND 2 | | | |  | | | Time: 2020-2023 | | | ( TITLE-ABS-KEY ( ( ( missed AND care ) OR ( missed AND nursing AND care ) OR ( unfinished AND nursing AND care ) OR ( unfinished AND care ) OR ( implicit AND rationing AND of AND nursing AND care ) OR ( implicit AND rationing ) OR ( rationing AND of AND nursing AND care ) OR ( rationed AND care ) OR ( prioritization AND process ) OR ( omitted AND nursing AND care ) OR ( task AND left AND undone ) OR ( task AND undone ) ) ) AND TITLE-ABS-KEY ( ( ( nurse ) OR ( nursing ) ) ) ) AND ( LIMIT-TO ( PUBYEAR , 2023 ) OR LIMIT-TO ( PUBYEAR , 2022 ) OR LIMIT-TO ( PUBYEAR , 2021 ) OR LIMIT-TO ( PUBYEAR , 2020 ) ) | 789 | | 01/01/2023  09:49 |
| 2 | | (TITLE-ABS-KEY (nurse) OR TITLE-ABS-KEY (nursing)) | | | |  | | | Time: 2020-2023 | | | (TITLE-ABS-KEY ( nurse ) OR TITLE-ABS-KEY ( nursing ) ) AND ( LIMIT-TO ( PUBYEAR , 2023 ) OR LIMIT-TO ( PUBYEAR , 2022 ) OR LIMIT-TO ( PUBYEAR , 2021 ) OR LIMIT-TO ( PUBYEAR , 2020 ) ) | 100,738 | | 01/01/2023  09:44 |
| 1 | | (TITLE-ABS-KEY (missed care) OR TITLE-ABS-KEY (missed nursing care) OR TITLE-ABS-KEY  (unfinished nursing care) OR TITLE-ABS-KEY (unfinished care) OR TITLE-ABS-KEY (implicit rationing of nursing care) OR TITLE-ABS-KEY (implicit rationing) OR TITLE-ABS-KEY (rationing of nursing care) OR TITLE-ABS-KEY (rationed care) OR TITLE-ABS-KEY (prioritization process) OR TITLE-ABS-KEY (omitted nursing care) OR TITLE-ABS-KEY (task left undone) OR TITLE-ABS-KEY (task undone) | | | |  | | | Time: 2020-2023 | | | ( TITLE-ABS-KEY ( missed AND care ) OR TITLE-ABS-KEY ( missed AND nursing AND care ) OR TITLE-ABS-KEY ( unfinished AND nursing AND care ) OR TITLE-ABS-KEY ( unfinished AND care ) OR TITLE-ABS-KEY ( implicit AND rationing AND of AND nursing AND care ) OR TITLE-ABS-KEY ( implicit AND rationing ) OR TITLE-ABS-KEY ( rationing AND of AND nursing AND care ) OR TITLE-ABS-KEY ( rationed AND care ) OR TITLE-ABS-KEY ( prioritization AND process ) OR TITLE-ABS-KEY ( omitted AND nursing AND care ) OR TITLE-ABS-KEY ( task AND left AND undone ) OR TITLE-ABS-KEY ( task AND undone ) ) AND ( LIMIT-TO ( PUBYEAR , 2023 ) OR LIMIT-TO ( PUBYEAR , 2022 ) OR LIMIT-TO ( PUBYEAR , 2021 ) OR LIMIT-TO ( PUBYEAR , 2020 ) ) | 8,380 | | 01/01/2023  09:42 |
