# Supplementary material for: Were the unfinished nursing care occurrence, reasons, and consequences different between COVID-19 and non-COVID-19 patients? A systematic review
Source: BMC Nurs. 2023 Sep 27;22:341. doi: 10.1186/s12912-023-01513-4 (PMC10523650; doi:10.1186/s12912-023-01513-4)
Supplement: Supplementary file 4 — Supplementary Material 4 [file 12912_2023_1513_MOESM4_ESM.docx]

**Supplementary Table 1.** Inclusion and exclusion criteria during the full texts screening process.

|  | **Inclusion Criteria** | **Exclusion Criteria** |
| --- | --- | --- |
| **Language** | English  Italian  Turkish | Others |
| **Design** | Comparative cross-sectional studies  Longitudinal studies  Cohort studies  Randomized controlled trials  Non-randomized controlled trials | Descriptive studies  Mix methods (quantitative-qualitative)  Methodologic studies  Editorials  Letters to the editor  Qualitative studies  Reviews  Commentaries  Book  Chapter of book  Book of congress or presentation |
| **Population** | Only nurses and nursing aides | Other healthcare workers* |
| **Setting** | All type of settings  All type of hospitals |  |
| **Outcome** | Reporting Unfinished Nursing Care, its reasons, and consequences | Not addressing any relevant topic of the inclusion criteria  Not addressing the reasons and consequences of Unfinished Nursing Care  Not addressing patients in the pandemic period  Not relevant topic  Not available abstract and full text |
| **Time frame** | Studies collecting data from January 1^st^, 2020 up to January 1^st^, 2023  Studies performed and collecting data before 2020 and compared with that collected during the pandemic in the same setting | Studies performed and collecting data before 2020 |
| **Instruments/tools** | Measuring Unfinished Nursing Care phenomenon (e.g., MISSCARE Survey, Unfinished Nursing Care Survey) | Measuring other phenomena (e.g., Fundamentals of Care) |

*If the study has been involved also nurses and shown the findings separately, there was considered the eligible data
